# Myocardial perfusion and oxidative metabolism in healthy subjects: sex-specific insights from vasodilatory stress ^11^C-acetate PET

**DOI:** 10.1186/s13550-025-01311-w

**Published:** 2025-09-02

**Authors:** Sang-Geon Cho, Hyung Yoon Kim, Ki Seong Park, Jahae Kim, Jang Bae Moon, Nuri Lee, Hyukjin Park, Jae Yeong Cho, Hyun Ju Yoon, Youngkeun Ahn, Joon-Hyung Doh, Eun-Seok Shin, Kye Hun Kim, Ho-Chun Song

**Affiliations:** 1 Department of Nuclear Medicine, Chonnam National University Hospital, Gwangju, Republic of Korea; 2 Department of Cardiovascular Medicine, Chonnam National University Hospital, Gwangju, Republic of Korea; 3 Division of Cardiology, College of Medicine, Inje University Ilsan-Paik Hospital, Goyang, Korea; 4 Department of Cardiology, Ulsan University Hospital, University of Ulsan College of Medicine, Ulsan, Republic of Korea; 5 Division of Cardiology, Department of Internal Medicine, Cardiovascular Center, Incheon Sejong Hospital, Incheon, Republic of Korea

**Keywords:** Myocardial perfusion, Oxidative metabolism, Sex-specific characteristics, Positron-Emission tomography (PET), Vasodilator stress, Reference values

## Abstract

**Background:**

^11^C-acetate positron emission tomography (PET) enables simultaneous quantification of myocardial blood flow (MBF) and oxidative metabolism; however, sex-specific normative data under vasodilator stress remain insufficiently defined. We aimed to characterize these parameters in healthy individuals.

**Results:**

Eighteen healthy individuals (9 males and 9 females, age/sex-matched; median age 43 years [range 34–65]) with normal echocardiography and no underlying cardiovascular disease underwent one-day rest-stress dynamic ^11^C-acetate PET with adenosine. Median stress MBF was 2.73 (2.24–3.11) mL/min/g and rest MBF 0.99 (0.82–1.14) mL/min/g. Stress kmono was 0.074 (0.063–0.082)/min, and rest kmono was 0.061 (0.050–0.068)/min. External cardiac work, total myocardial oxygen consumption (MVO₂), and MEE were 4.24 (3.75–5.54) × 10⁵ mL·mmHg/min, 16.5 (14.9–20.9) mL/min, and 17.7% (14.0–24.2%), respectively. Compared to males, females exhibited significantly higher corrected rest MBF (1.14 vs. 0.96 mL/min/g, *p* = 0.032), stress kmono (0.078 vs. 0.070 /min, *p* = 0.005), and total MVO₂ (17.6 vs. 15.5 mL/min, *p* = 0.003). Despite similar external cardiac work, myocardial external efficiency was significantly lower in females (15.5% vs. 20.3%, *p* = 0.009). MBF and oxidative metabolism were positively correlated in both sexes.

**Conclusions:**

This study assesses reference ranges and demonstrates significant sex-based differences in myocardial perfusion and oxidative metabolism using vasodilatory stress ^11^C-acetate PET. These findings provide a valuable physiological framework for assessing perfusion-metabolism abnormalities in clinical practice.

**Supplementary Information:**

The online version contains supplementary material available at 10.1186/s13550-025-01311-w.

## Introduction

The pathophysiology of coronary artery disease (CAD) has shown substantial differences between the sexes. Characteristically, females tend to present more angina symptoms while are less likely to have obstructive CAD [1, 2]. Clinical studies provide plausible explanations for such phenomena by showing the inherited differences in myocardial metabolism, indicating that oxidative metabolism (especially free fatty acid oxidation) is more pronounced in females [3, 4], which contributes to increased oxygen demand.

To noninvasively assess the sex-specific characteristics of myocardial metabolism, dynamic ^11^C-acetate cardiac positron emission tomography (PET) has been utilized. It can quantify the mitochondrial oxidative metabolism by measuring the washout rate of ^11^C-acetate from the myocardium. In addition, myocardial blood flow (MBF) can be quantified based on the compartment model specifically designed for ^11^C-acetate PET [5]. It provides quantitative metrics to establish the diagnosis and assess the severity coronary microvascular dysfunction, which is a particularly common pathophysiology in females [6–8]. Thus, ^11^C-acetate PET can be a powerful tool that can simultaneously evaluate the abnormalities in both myocardial perfusion and metabolism in patients with various cardiac disease entities [4, 9, 10] as well as CAD.

However, existing data on the normal ranges of MBF and oxidative metabolism are limited to resting state or inotropic stress (e.g., exercise stress or dobutamine stress) [3, 9–17]; those required to set normal ranges under vasodilatory stress testing — which are frequently used for cardiac PET imaging in CAD [18–20] — are lacking. Accordingly, we aim to evaluate reference ranges of MBF and myocardial oxidative metabolism under vasodilatory stress in healthy individuals and to explore sex-specific physiological differences with potential clinical relevance.

## Materials and methods

### Study design and patients

Healthy adults were prospectively recruited via poster advertisement. The inclusion criteria were age of 20–65 years old; no history of acute or chronic coronary syndrome; no known structural cardiac diseases; no underlying disease requiring medication. Exclusion criteria included abnormal echocardiography or poor echocardiographic window; contraindication to adenosine vasodilator stress testing; irregular heartbeat; other systemic conditions potentially affecting cardiac function. An experienced cardiologist of > 10 year-experience (H.Y. K.) screened the participants regarding the health status and whether they fulfill the study criteria. Following the initial recruitment and echocardiographic screening, the final eighteen participants were screened through age-matching between females and males. All participants were asked to sign the consent upon a detailed study explanation. The health status of each participant was confirmed based on a standardized checklist, including: (1) no prior history of cardiovascular, metabolic, or systemic diseases; (2) normal findings on physical examination; (3) normal resting echocardiography with preserved LVEF and no structural abnormalities.

### 2D Echocardiography and speckle tracking echocardiography

Comprehensive transthoracic echocardiography (M-mode, 2-D, and Doppler) was performed using commercially available equipment (Vivid E95, GE Medical system, Milwaukee, WI). Left ventricular (LV) chamber size and wall thickness were measured by using 2016 American Society of Echocardiography (ASE)’s guideline and standards [21]. LV mass was calculated using the conventional cube formula and LV hypertrophy (LVH) was determined according to the ASE’s chamber quantitation guideline (> 95 g/m^2^ for women, >115 g/m^2^ for men) [21]. Analysis of the 2D STE images was performed with a software package (EchoPAC, GE Ultrasound, Haifa, Israel by an experienced investigator blinded to all clinical information of the enrolled subjects. Peak longitudinal strain was computed automatically for each LV segments and averaged value was reported as global strain.

### Cardiac ^11^C-acetate PET

For the stress testing for ^11^C-acetate PET, the patients abstained of caffeine-containing foods or beverages at least 3 days from the PET study. Vasodilatory medication potentially affecting adenosine stress was also stopped for the corresponding period. Two intravenous lines, which were used for radiopharmaceutical injection and adenosine infusion, respectively, were inserted into the patient’s arms. After the patient lay down in a supine position, attenuation correction computed tomography (CT) was acquired. Resting PET images were obtained first, which simultaneously began with the intravenous ^11^C-acetate injection by an autoinjector. Adenosine stress, which lasted for 6 min, began immediately following the 20-minute dynamic resting PET acquisition. At 3 min of adenosine infusion, which lasted for 6 min, stress dose of ^11^C-acetate was injected. The ^11^C-acetate dosage and detailed dynamic image acquisition methodologies were as detailed previously [22].

### Quantification of myocardial blood flow and oxidative metabolism

From the rest and stress PET images, MBF was quantified based on the one-compartment model suggested by van den Hoff et al. [5], which showed the best agreement with O-15 water PET [17]. The ratio of stress MBF to rest MBF was defined as myocardial flow reserve (MFR). Using the downslope segment of the time-activity curve of ^11^C-acetate PET (≥ 2 min), the rate of washout was quantified (k_mono_) in the unit of /min. We also calculated k_mono_ reserve, which was defined as the ratio of stress k_mono_ to rest k_mono_ [22]. An analytic software package (Carimas™, Turku PET Centre, Finland) dedicated to quantifying dynamic PET parameters was utilized.

Oxygen consumption (MVO_2_) was calculated by the formula suggested by previous reports [9, 10]. MVO_2_ was multiplied by LV mass (measured by echocardiography) to obtain total MVO_2_.

### Calculation of myocardial work and efficiency

External cardiac work was calculated by multiplying end-systolic pressure (ESP), stroke volume (SV), and heart rate (HR) (mmHg × mL/min). To convert EW into Joules (J per minute), the result was multiplied by a factor of 1.33 × 10^− 4^. Myocardial external efficiency (MEE) was calculated in percentage by dividing the externally measurable cardiac work with total MVO_2_, as previously described [9, 10]. Resting LV myocardial mass and SV were derived from echocardiography, which was performed within 1 week of a specific ^11^C-acetate PET.

### Statistical analyses

Echocardiographic and PET parameters were compared between the groups along with demographic and clinical characteristics. Regarding continuous variables obtained from the imaging studies, mutual correlations were also analyzed. The comparisons of averages were performed based on either student’s t-test or Mann-Whitney U-test according to the data distribution assessed by Shapiro-Wilk normality test. Correlation analyses were performed either by Pearson’s correlation test or Spearman’s rank correlation test. Multiple linear regression was performed to assess the factor affecting imaging metrics. Statistical analyses were performed using R statistical package (version 4.4.2). A p-value < 0.05 was considered statistically significant.

## Results

### Baseline characteristics

The baseline characteristics of the study participants are summarized in Table 1. The median age of the participants was 43 years (interquartile range [IQR] 40–51), with a total range of 34 to 66 years. No difference was found for age between sexes. Females had significantly smaller height and body weight, which resulted in smaller body surface area. On echocardiography, females showed significantly lower diastolic interventricular septal thickness (IVSd), posterior wall thickness (LVPWd), and relative well thickness (RWT). LV mass or LV mass index tended to be higher for males but were not significantly different. Among the diastolic parameters, E velocity was significantly higher in females (*p* = 0.005). No significant difference was found for systolic function (e.g., LV ejection fraction, global longitudinal strain [GLS]). The comparisons regarding demographic and echocardiographic measurements between sexes are summarized in Table 1.

**Table 1:** Demographic and echocardiographic characteristics of study participants by sex

	Total (*n* = 18)	Females (*n* = 9)	Males (*n* = 9)
**Demographics**			
Age (years)	43 (IQR 40–51), range 34–66	43 (39–48), 34–66	43 (41–56), 35–64
Height (cm)	165 (161–170)	161 (159–164)*	170 (170–173)
Body weight (kg)	71 (59–78)	59 (53–72)*	74 (70–85)
Body mass index (kg/m^2^)	24.0 (21.8–28.5)	22.1 (21.7–26.8)	25.3 (23.5–28.7)
Body surface area (m^2^)	1.80 (1.64–1.92)	1.64 (1.52–1.78)*	1.89 (1.83–1.97)
***Echocardiography***			
LVIDd (mm)	49 (44–52)	49 (46–52)	44 (42–52)
LVIDs (mm)	30 (25–33)	31 (29–33)	28 (25–31)
IVSd (mm)	8 (7–9)	6.9 (6.7–7.5)*	8.3 (7.9–8.9)
LVPWd (mm)	7 (7–9)	6.5 (6.4–7.4)*	8.3 (7.7–8.8)
TR Vmax (m/s)	2.11 (1.93–2.25)	2.04 (1.82–2.18)	2.14 (2.00–2.38)
E velocity (m/s)	0.67 (0.56–0.75)	0.75 (0.70–0.87)*	0.57 (0.55–0.65)
A velocity (m/s)	0.60 (0.52–0.77)	0.55 (0.39–0.80)	0.62 (0.57–0.69)
Septal E’ (m/s)	0.11 (0.08–0.12)	0.12 (0.09–0.14)	0.10 (0.08–0.11)
E/septal E’	6.12 (5.45–7.01)	6.2 (5.5–9.3)	6.1 (5.8–6.9)
DT (ms)	191 (170–222)	200.6 (173.2–223.2)	189.1 (163.7–196.7)
LV mass (g)	114 (97–139)	109 (97–114)	117 (103–150)
LVMI (g/m^2^)	64 (55–72)	63.4 (58.1–68.3)	64.1 (54.9–75.5)
RWT	0.31 (0.28–0.38)	0.28 (0.26–0.30)*	0.37 (0.32–0.40)
LVEF (%)	69 (62–73)	66 (62–73)	70 (63–73)
|GLS (%)|	-19.1 (-19.9–-17.9)	19.1 (18.8–20.3)	18.9 (17.1–19.6)
Stroke volume (mL)	65 (57–82)	66.1 (57.4–80.0)	63.2 (60.6–82.1)
***Hemodynamics at PET acquisition***			
***Stress***			
Systolic BP (mmHg)	134 (120–144)	127 (121–138)	134 (122–137)
Diastolic BP (mmHg)	71 (62–81)	65 (60–73)	71 (64–81)
HR (/min)	88 (75–99)	92 (87–96)**	88 (80–97)**
RPP (mmHg/min)	11,834 (9424–13969)	12,192 (10080–13500)**	11,834 (10080–13600)**
***Rest***			
Systolic BP (mmHg)	133 (128–153)	136 (130–141)	133 (131–148)
Diastolic BP (mmHg)	75 (70–83)	72 (66–74)	75 (71–81)
HR (/min)	63 (62–75)	66 (61–73)	63 (62–74)
RPP (mmHg/min)	9324 (8418–10232)	9384 (8712–10478)	9324 (8584–9956)

Values are given as mean ± standard deviation or median (interquartile range) according to the data distribution

BP, blood pressure; DT, deceleration time; GLS, global longitudinal strain; IVSd, end-diastolic interventricular septal thickness; LV, left ventricular; LVEF, left ventricular ejection fraction; LVIDd, end-diastolic left ventricular internal diameter; LVIDs, end-systolic left ventricular internal diameter; LVMI, left ventricular mass index; RWT, relative wall thickness; TR Vmax, tricuspid valve regurgitation peak velocity

RPP was calculated by multiplying SBP*HR; corrected rest MBF was calculated by dividing rest MBF with rest RPP; corrected MFR was calculated by dividing stress MBF with corrected rest MBF. Values are given as mean ± standard deviation or median (interquartile range) according to the data distribution. *p < 0.05 vs. males, **p < 0.05 vs. rest

### Normal ranges of myocardial blood flow and oxidative metabolism and sex differences

Table 2 presents the values of MBF and oxidative metabolism parameters measured by vasodilatory stress ^11^C-acetate PET in eighteen healthy individuals. Females exhibited higher stress and rest MBF, with borderline statistical significance. After correction for rate-pressure product (RPP), rest MBF was significantly higher in females. MFR did not differ between sexes, regardless of RPP correction. Resting and stress hemodynamics (BP, heart rate, and RPP) were comparable between groups (Table 1).

**Table 2:** Myocardial perfusion and oxidative metabolism measured by ^11^C-acetate PET in healthy subjects

	Total (*n* = 18)	Females (*n* = 9)	Males (*n* = 9)	*p*
Stress MBF (mL/min/g)	2.73 (2.24–3.11)	3.11 (2.44–3.66)	2.73 (2.24–3.11)	0.093
Rest MBF (mL/min/g)	0.99 (0.82–1.14)	1.02 (0.96–1.14)	0.82 (0.66–1.02)	0.051
Corrected rest MBF (mL/min/g)	1.07 (0.86–1.14)	1.14 (1.07–1.21)*	0.97 (0.78–1.02)	0.013
MFR	3.01 (2.36–3.46)	2.98 (2.18–3.59)	3.03 (2.62–3.28)	0.605
Corrected MFR	2.81 (2.35–3.20)	2.80 (2.07–3.20)	2.95 (2.51–3.23)	0.480
Stress k_mono_ (/min)	0.074 (0.063–0.082)	0.082 (0.081–0.093)*	0.063 (0.051–0.067)	< 0.001
Rest k_mono_ (/min)	0.061 (0.050–0.068)	0.068 (0.065–0.074)*	0.050 (0.042–0.054)	0.005
k_mono_ reserve	1.26 (1.12–1.29)	1.21 (1.12–1.58)	1.26 (1.20–1.29)	0.930
Total MVO_2_ (mL/min)	16.5 (14.9–20.9)	20.2 (16.4–21.2)*	14.9 (12.6–16.1)	0.014
External cardiac work (*10^5^ mL*mmHg/min)	4.24 (3.75–5.54)	4.20 (3.75–4.93)	4.51 (3.86–5.54)	1.000
MEE (%)	17.7 (14.0–24.2)	14.7 (12.8–16.3)*	20.2 (19.4–29.5)	0.022

*p < 0.05 vs. males

Values are given as mean ± standard deviation or median (interquartile range) according to the data distribution

kmono, mono-exponentially fitted myocardial ^11^C-acetate clearance rate (oxidative metabolism); MBF, myocardial blood flow; MEE, myocardial external efficiency; MFR, myocardial flow reserve; MVO2, myocardial oxygen consumption rate; PET, positron emission tomography

Regarding oxidative metabolism, k_mono_ increased by a median of 0.013 (25%) during adenosine stress, with no significant difference in the degree of increase between females (0.012, 21%) and males (0.014, 26%). Notably, females demonstrated consistently higher k_mono_ values at both rest and stress, with non-overlapping interquartile ranges. This resulted in significantly higher total MVO_2_ in females, despite having a lower median LV mass (109 g vs. 117 g; *p* = 0.112). Notably, myocardial external efficiency (MEE) was significantly lower in females, despite similar external cardiac work (Table 2).

### Correlations of MBF and oxidative metabolism with demographics and echocardiographic indices

**Fig. 1:**
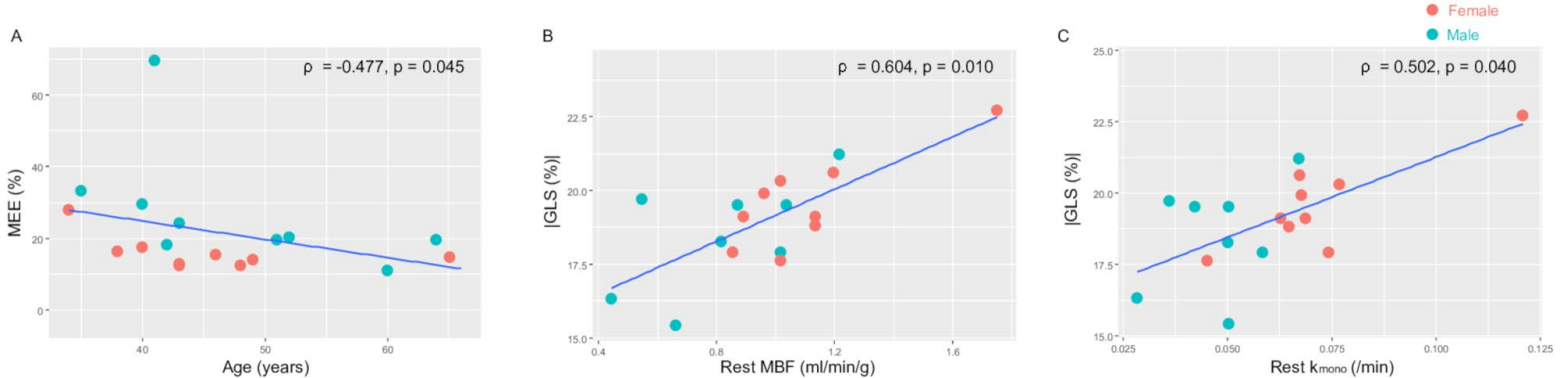
Correlation of ^11^C-acetate PET parameters with age and LV strain. Age and MEE showed a negative linear correlation (**A**). Rest MBF (**B**) and k_mono_ (C) positively correlated with the absolute value of GLS (|GLS|). GLS, global longitudinal strain; k_mono_, mono-exponentially fitted myocardial ^11^C-acetate clearance rate (oxidative metabolism); LV, left ventricular; MBF, myocardial blood flow; MEE, myocardial external efficiency; PET, positron emission tomography

There was a modest negative linear correlation between age and MEE (ρ = 0.477, *p* = 0.045) (Fig. 1A). In contrast, age showed no significant correlations with MBF, MFR, k_mono_, k_mono_ reserve, or hemodynamics during PET acquisition.

Among echocardiographic LV function indices, the absolute value of GLS (|GLS|) demonstrated significant positive correlations with rest MBF and rest k_mono_ (Fig. 1B-C). These correlations remained significant even after correction of rest MBF by RPP (ρ = 0.513, *p* = 0.035).

Interestingly, |GLS| did not correlate with stress MBF or stress k_mono_. Instead, inverse relationship were observed between |GLS| vs. MFR (ρ = -0.578, *p* = 0.015), as well as between k_mono_ reserve (ρ = -0.625, *p* = 0.007).

### Correlations among MBF, oxidative metabolism, and hemodynamics

**Fig. 2:**
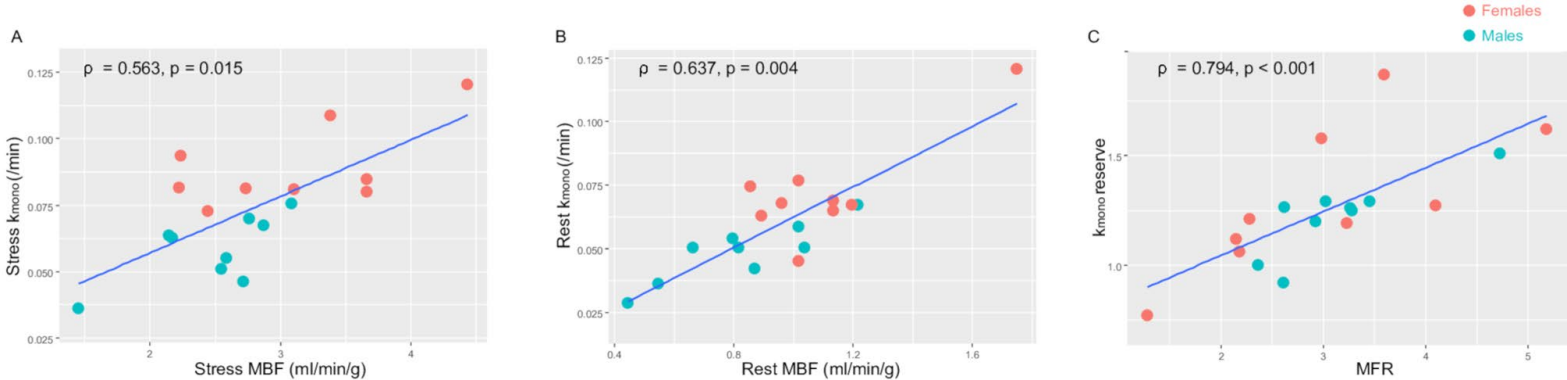
Correlations among myocardial perfusion and oxidative metabolism. MBF and k_mono_ showed significant positive linear correlations both during stress and at rest (**A-B**). Stress-to-rest ratios of these metrics (MFR vs. k_mono_ reserve) also showed a significant positive linear correlation (**C**). MFR, myocardial flow reserve

Among PET parameters, MBF and k_mono_ showed significant positive correlations at both rest and stress conditions. Additionally, MFR and k_mono_ reserve were also positively correlated (Fig. 2). Importantly, these relationships remained significant after the correction of rest MBF by RPP: corrected rest MBF vs. rest k_mono_ (ρ = 0.550, *p* = 0.018); corrected MFR vs. k_mono_ reserve (ρ = 0. 731, *p* < 0.001).

Hemodynamic parameters during PET acquisition also influenced these findings. RPP during stress and at rest significantly correlated with stress MBF (ρ = 0.618, *p* = 0.006) and rest k_mono_ (ρ = 0.473, *p* = 0.047), respectively. This correlation appeared to be primarily driven by HR, as no significant association was found with systolic or diastolic BP. Furthermore, the stress-to-rest ratio of RPP significantly correlated with both MFR (ρ = 0.620, *p* = 0.007) and k_mono_ reserve (ρ = 0.596, *p* = 0.009). Interestingly, unlike the MBF and k_mono_ correlations, reserve parameters demonstrated significant associations with BP rather than HR (Supplementary Table 1).

### Factors affecting LV function and oxidative metabolism

Multiple linear regression analyses were performed to assess factors associated with LV function (|GLS|), myocardial oxidative metabolism (rest k_mono_), and its efficiency (MEE). The analysis revealed that rest MBF was more closely associated (β = 0.657, *p* = 0.079) with |GLS| than rest k_mono_ (β = 0.097, *p* = 0.783). Both sex (β = -0.551, *p* = 0.003 for males) and rest RPP (β = 0.536, *p* = 0.003) were significant independent predictors of rest k_mono_ in the multivariate analysis. Notably, sex (β = 0.483, *p* = 0.036 for males) demonstrated a stronger association with MEE compared to age (β = -0.413, *p* = 0.068).

## Discussion

This study evaluated sex-specific reference ranges for MBF and myocardial oxidative metabolism in healthy individuals using vasodilatory stress ^11^C-acetate PET. Adenosine stress induced > 20% increase of oxidative metabolism, which correlated with that in myocardial perfusion. Females demonstrated higher oxidative metabolism and lower metabolic efficiency (approximately 73% of that of males). MBF and oxidative metabolism significantly correlated with each other.

**Fig. 3:**
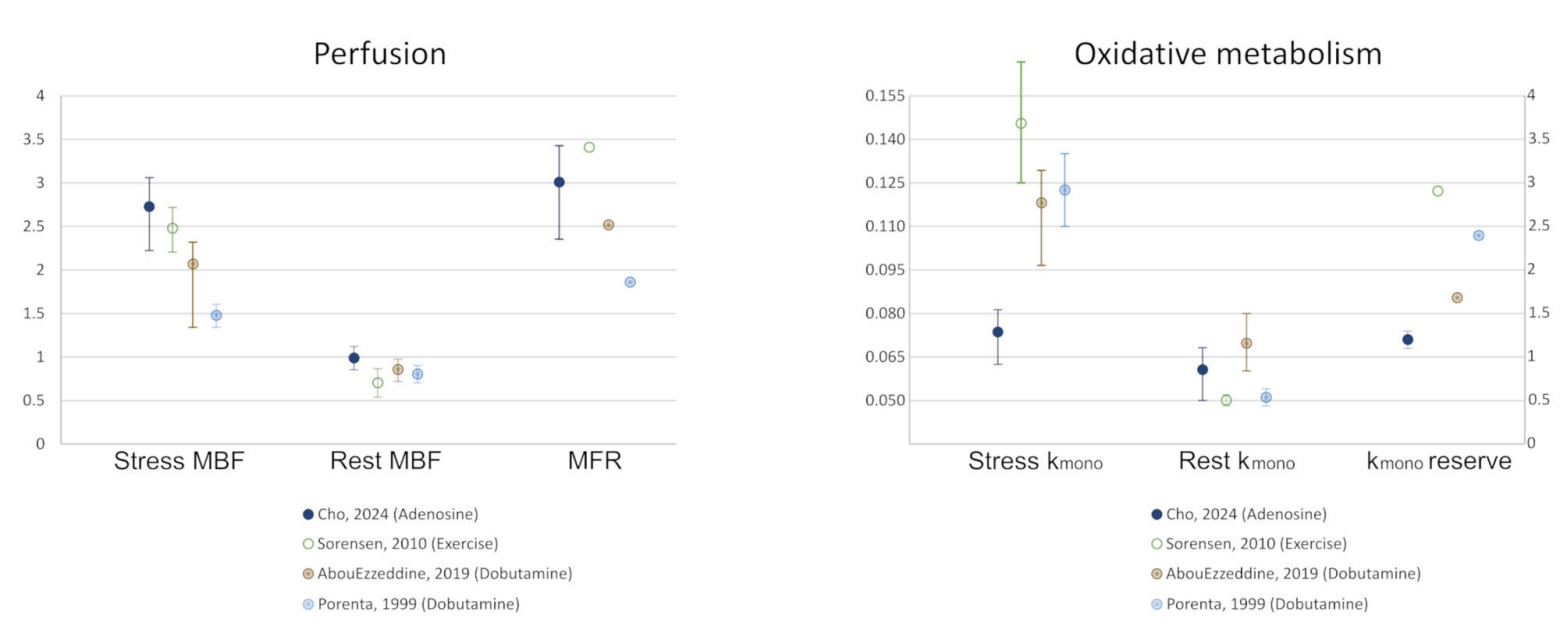
Comparison of myocardial perfusion and oxidative metabolism among studies with available stress measurements. The mark and error bars represent median with interquartile range or mean ± standard deviation. The MBF values of AbouEzzedine et al. [10] were derived using the median LV mass from the study data; the k_mono_ values of Porenta et al. [12] were derived from the equation for calculating MVO_2_. MFR values from the previous studies were calculated by dividing the representative stress MBF with rest MBF values (median or mean); the error bars of MFR could not be expressed due to lack of data. Error bars indicate standard deviation or (interquartile) ranges according to study statistics

In comparison with previously published data on normal ranges of ^11^C-acetate PET parameters, our findings demonstrate comparable values for resting MBF and k_mono_ (Supplementary Table 2). However, significant discrepancies are observed in stress-induced responses. As shown in Fig. 3, when the data from the present (closed blue dot) and previous studies with available stress MBF and k_mono_ values [10–12], dobutamine and exercise stress testing seems to induce a similar or lesser extent of increase in MBF but a much more remarkable increase in oxidative metabolism. This suggests that dobutamine and exercise stress drastically increase myocardial oxygen demand but may not provide sufficient blood supply to match the demand, that can be critically risky to some conditions [23]. Also, this probably contributes to the superior efficacy of dobutamine stress testing in the evaluation of stress-induced wall motion abnormality [24], but relatively inferior efficacy to induce flow heterogeneity in myocardial perfusion imaging [25]. These observations underscore the distinct hemodynamic responses between stress modalities, highlighting the need for modality-specific reference ranges. Our study provides a unique normal dataset of vasodilator stress ^11^C-acetate PET to simultaneously assess vasodilatory capacity relative to oxygen demand in patients with various cardiac diseases. Interestingly, the increase in k_mono_ by adenosine stress was only 25% of the resting state, similar to patients with recent acute ST-segment elevation myocardial infarction (*n* = 30, 77% males, median 3 days post-infarction) [22]. In that study, the median value of k_mono_ reserve (referred to as oxidative metabolism reserve [OMR]) was almost identical to the present study, but with a wider distribution (1.25 [0.87–1.74]). It did not differ between infarct-related artery territory (IRAs) vs. non-IRA territory or non-IRA territories with vs. without significant stenosis.

The sex differences in oxidative metabolism were clearly recognized in the present study, consistent with the previous reports [26, 27]. Female hearts are considered to consume more oxygen, reportedly due to different substrate preference in energy production during the resting state [3]. Such differences also exist in the non-cardiac skeletal muscles; the enzymatic and genetic studies in the limb muscles revealed greater capacity of beta-oxidation and lower glycolytic potential [28, 29]. Oxidative metabolism has been shown to tightly correlate with MBF [3, 12]. It was reproduced in our data and further extrapolated to vasodilatory stress as well. The increase in blood flow is essential for the myocardium to match the oxygen demand (e.g., tachycardia). Adenosine stress caused increase in HR and RPP, which paralleled with increased k_mono_ by 25% and about 3-fold increase in MBF. Rest MBF was higher in females and even statistically more significant after RPP correction. Similar results were observed by Duvernoy et al. [4], which was especially remarkable among diabetic subjects. Even with similar amount of external cardiac workload, females may have much higher oxidative metabolism demanding incremental amount of oxygen (≈ blood flow) relative to males, as reflected in a markedly lower MEE of females relative to males. It provides plausible explanations regarding the vulnerability of females to anginal symptoms and adverse cardiac events despite milder degrees of CAD as compared to males.

These physiological differences in myocardial perfusion and metabolism between sexes offer critical insight into observed disparities in invasive physiological indices and clinical outcomes. For example, women with deferred revascularization based on an fractional flow reserve (FFR) > 0.80 have shown higher rates of adverse events compared to men [30], underscoring that epicardial-focused assessment may overlook microvascular and metabolic factors. The higher resting MBF and oxidative metabolism observed in our study provide a pathophysiological rationale for this discrepancy, suggesting that female myocardium’s elevated baseline demand may reduce the reliability of FFR-based decision-making. Likewise, lower CFR values in women, often attributed to higher resting coronary flow rather than true impairment [31], are consistent with our findings. This supports interpreting reduced CFR in women as a physiologic adaptation rather than pathology. The role of index of microcirculatory resistance (IMR) in sex differences remains debated [32]. Our demonstration of higher resting myocardial demand and lower efficiency in females offers compelling mechanistic support for the variability observed in IMR studies, indicating that sex-specific metabolic profiles may predispose women to microvascular resistance elevations. Together, these findings highlight the essential role of sex-specific physiology in interpreting invasive indices and underscore the unique contribution of our study in providing the metabolic background for these observed clinical discrepancies.

### Study limitations

The current study analyzed only adenosine vasodilatory stress testing, which is most commonly used for cardiac PET. However, the vasodilatory agents (e.g., regadenoson, dipyridamole) for cardiac PET varies by countries, which may limit the generalizability of the data. There were elderly participants with age > 60 years old in our study, who could have high likelihood of subclinical CAD. However, all their stress MBF were above 2.17 mL/min/g (all MFR > 2.0), corresponding to ‘excellent coronary flow capacity’ suggested by Gould et al. [33]. The information on menstrual phase was not available for the in premenopausal women among the participants of the present study. However, it was not found significantly affect oxygen consumption in a previous study by Peterson et al. [3]. Additionally, although this study focused on healthy individuals, extrapolation to patient populations requires caution. Finally, the relatively small sample size of eighteen participants may limit the statistical power and generalizability of our findings. Nevertheless, this sample size is comparable to that of prior studies utilizing dynamic ^11^C-acetate PET in healthy individuals, and our study provides important exploratory data for establishing sex-specific reference values and physiological insights that can guide future larger-scale investigations.

## Conclusion

In conclusion, this study provides preliminary normal reference ranges of MBF and oxidative metabolism under vasodilatory stress ^11^C-acetate PET in healthy individuals. Importantly, it suggests potential sex-specific differences, with females exhibiting higher baseline myocardial oxygen demand and lower metabolic efficiency. These physiological disparities provide critical insight into observed sex-based variations in invasive and non-invasive coronary physiological indices and support the importance of sex-tailored interpretation in clinical decision-making. Further large-scale studies are warranted to validate the clinical impact of integrating sex-specific metabolic profiles into patient evaluation and risk stratification strategies.

## Supplementary Information

Below is the link to the electronic supplementary material.

Supplementary Material 1

## Data Availability

The datasets generated and analyzed during the current study are available from the corresponding author on reasonable request.
