# Supplementary material for: Myocardial perfusion and oxidative metabolism in healthy subjects: sex-specific insights from vasodilatory stress 11C-acetate PET
Source: EJNMMI Res. 2025 Sep 2;15:116. doi: 10.1186/s13550-025-01311-w (PMC12405121; doi:10.1186/s13550-025-01311-w)
Supplement: Supplementary file 1 — Supplementary Material 1 [file 13550_2025_1311_MOESM1_ESM.docx]

**Supplementary Table 1. Correlations between hemodynamic and PET parameters**

|  |  | **Corresponding hemodynamics** | | | |
| --- | --- | --- | --- | --- | --- |
|  |  | **Systolic BP (mmHg)** | **Diastolic BP**  **(mmHg)** | **HR**  **(/min)** | **RPP**  **(mmHg/min)** |
| **Stress** | **MBF** | 0.307 | 0.308 | 0.589* | 0.618** |
|  | **k_mono_ (/min)** | 0.205 | -0.174 | 0.314 | 0.385 |
| **Rest** | **MBF** | -0.077 | -0.348 | 0.474* | 0.380 |
|  | **k_mono_ (/min)** | 0.074 | -0.341 | 0.467 | 0.473* |
|  |  | **Stress/rest ratios of hemodynamics** | | | |
|  |  | **Systolic BP** | **Diastolic BP** | **HR** | **RPP** |
| **MFR** | | 0.531* | 0.490* | 0.461 | 0.620** |
| **k_mono_ reserve** | | 0.587* | 0.355 | 0.394 | 0.596** |

The values represent correlation coefficients based on Spearman’s correlation analysis.

BP, blood pressure; HR, heart rate; k_mono_, mono-exponentially fitted myocardial ^11^C-acetate clearance rate (oxidative metabolism); MBF, myocardial blood flow; MFR, myocardial flow reserve. *p < 0.05; **p < 0.01.

**Supplementary Table 2. Ranges of simultaneously measured myocardial perfusion and oxidative metabolism measured by ^11^C-acetate PET in healthy subjects from previous literature.**

| **Studies** | **Number of subjects (n), sex, and age** | **Perfusion** | | | **Oxidative metabolism at rest** | | |
| --- | --- | --- | --- | --- | --- | --- | --- |
|  |  | Parameter | Normal range | Female vs. male | Parameter | Normal range | Female vs. male |
| Porenta et al.  [12] | 11  (F = 1, M = 10)  Mean, 23 yo (range, 19–31) | Stress MBF (Dob) | 1.48 ± 0.15 | N/A | k_mono_ | N/A | N/A |
|  |  | Rest MBF | 0.80 ± 0.06 | N/A | MVO_2_ | 88 ± 15 µL/min/g | N/A |
|  |  | MFR | N/A | N/A | MEE | 16 ± 6 | N/A |
| Güçlü et al.  [13] | 14  (F = 5, M = 9)  48 ± 11 yo | Stress MBF | N/A | N/A | k_2_ | 0.08 ± 0.02/min | N/A |
|  |  | Rest MBF | N/A | N/A | MVO_2_ | 1.43 ± 0.27 mL/beat/g*10^-3^ | N/A |
|  |  | MFR | N/A | N/A | MEE | 42 ± 6 | N/A |
| Hansson et al.  [14] | 10  (no data on sex composition)  63 ± 4 yo | Stress MBF | N/A | N/A | k_mono_  k_2_ | 0.062 ± 0.009  0.083 ± 0.015 | N/A |
|  |  | Rest MBF | N/A | N/A | MVO_2_ | N/A | N/A |
|  |  | MFR | N/A | N/A | MEE** | 11.8 ± 2.9 | N/A |
| Peterson et al.  [3] | 25  (F = 13, M = 12)  Range, 20–44 yo | Stress MBF | N/A | N/A | k_mono_ | N/A | N/A |
|  |  | Rest MBF  (H_2_^15^O) | F, 1.13 ± 0.27  M, 0.99 ± 0.19 | p = 0.16 | MVO_2_ | N/A (figure only) | p < 0.005 |
|  |  | MFR | N/A | N/A | MEE | F, 12.4 ± 2.3  M, 14.6 ± 3.4 | p = 0.07 |
| Sörensen et al.  [11] | 5  (athletes, no data on sex composition or age) | Stress MBF (Ex) | 2.48 ± 0.25 | N/A | k_mono_  k_2_ | 0.050 ± 0.005  0.057 ± 0.011 | N/A |
|  |  | Rest MBF | 0.71 ± 0.17 | N/A | MVO_2_ | N/A | N/A |
|  |  | MFR | N/A | N/A | MEE | N/A | N/A |
| Timmer et al.  [17] | 18  (F = 10, M = 8)  44 ± 15 yo | Stress MBF | N/A | N/A | k_mono_ | N/A | N/A |
|  |  | Rest MBF | 0.88 ± 0.25*** | N/A | MVO_2_ | N/A | N/A |
|  |  | MFR | N/A | N/A | MEE | N/A | N/A |
| Sciacca et al.  [15] | 8  (F = 4, M = 4)  42 ± 8 yo | Stress MBF | N/A | N/A | k_mono_ | N/A | N/A |
|  |  | Rest MBF | 1.06 ± 0.25 | N/A | MVO_2_ | N/A | N/A |
|  |  | MFR | N/A | N/A | MEE | N/A | N/A |
| Sun et al.  [16] | 12  (F = 3, M = 9)  26 ± 6 yo | Stress MBF | N/A | N/A | k_2_ | 0.056 ± 0.017 | N/A |
|  |  | Rest MBF | 0.70 ± 0.11 | N/A | MVO_2_ | 8.2 ± 2.1 mL/100 g/min | N/A |
|  |  | MFR | N/A | N/A | MEE | N/A | N/A |
| AbouEzzeddine et al.  [10] | 19  (F = 13, M = 6)  70 (67–78) yo | Stress MBF (Dob) | 2.07† | N/A | k_mono_ | 0.070 (0.059–0.080) | N/A |
|  |  | Rest MBF | 0.82† | N/A | Total MVO_2_ | 25.2 (20.0–30.7) mL/min | N/A |
|  |  | MFR | 2.52† | N/A | MEE | 14.2 (10.0–17.1) | N/A |
| Shi et al.  [9] | 12  (F = 0, M = 12)  50.3 (31–65) yo | Stress MBF | N/A | N/A | k_1_  k_2_  k_mono_ | 0.715 ± 0.120/min  0.114 ± 0.017/min  0.066 ± 0.007/min | N/A |
|  |  | Rest MBF | 0.820 ± 0.184 | N/A | MVO_2_ | 0.144 ±  0.023 mL/min/g | N/A |
|  |  | MFR | N/A | N/A | MEE | 22.4 ± 4.6 | N/A |

Stress and rest MBF are given as mL/min/g; k_mono_, k_1_, and k_2_ are given as /min; MEE is given as %. The units of MVO_2_ values are given individually as they are provided with variable units in the literature.
